# Dr Frank Marcus (1928–2022)

**DOI:** 10.1007/s12471-023-01774-3

**Published:** 2023-03-20

**Authors:** Richard N. W. Hauer

**Affiliations:** Department of Cardiology, University Medical Centre Utrecht, Utrecht, The Netherlands

The American Dr Frank Marcus passed away in December 2022, at the age of 94. He was a giant in cardiology, who had highly original thoughts and numerous key publications in the field of cardiac arrhythmias.

Marcus was born in Haverstraw, New York on March 23, 1928. He received his BA degree from Columbia University, his MS degree in physiology from Tufts University and his MD degree from Boston University. He was trained in internal medicine at the Peter Bent Brigham Hospital in Boston and spent a year as a research fellow at the Brigham. This was followed by a training in cardiology at Georgetown University Hospital in Washington. He became Chief Medical Resident at Georgetown and Chief of Cardiology at the same institution. In 1969, he was appointed Chief of Cardiology and Professor of Medicine at the University of Arizona in Tucson. In 1982, he was appointed Distinguished Professor of Medicine and Director of the Arrhythmia Service and finally, in 1999, as Emeritus Professor at the same university, but he continued his clinical and research activities up to his retirement in June 2020. Among his honours are the Outstanding Achievement Award of the European Cardiac Arrhythmia Society and the Pioneer in Cardiac Pacing and Electrophysiology Award of the Heart Rhythm Society, both in 2011.

His early research activities focused on the field of pharmacology, particularly on digitalis. However, a sabbatical at the Jean Rostand Hospital in Paris in 1979, with Guy Fontaine and Yves Grosgogeat as mentors, paved the way for two—at that time—completely new challenges: catheter ablation and arrhythmogenic right ventricular cardiomyopathy (ARVC). Catheter ablation was performed with direct current energy, delivered by an external defibrillator connected to a venous catheter with an endocardially positioned terminal current delivery electrode. This method has important side effects and requires anaesthesia. These drawbacks prompted Marcus, while being back in Arizona, to explore radiofrequency (RF) energy for ablation in animal models [1]. Not so many people realise that it was Marcus who actually started RF ablation, a presently worldwide used, safe methodology. Studying ARVC patients in Paris gave rise to the first systematic description of this disease in 1982, including diagnostic features and awareness of its familiar occurrence [2]. Since then, ARVC remained his primary research focus, with many original contributions culminating in the revised internationally accepted consensus-based task force criteria for ARVC diagnosis, which were published in the *European Heart Journal* and *Circulation* in 2010 [3]. This work was clinically relevant and facilitated meaningful comparison of scientific results.

Being aware of the breakthrough ARVC publication in 1982, it felt as a privilege to meet Marcus in person at the American Heart Association convention in Miami in 1984, and in 1986, I visited him in his hometown Tucson. In 1987, while he and his wife Janet were on a sportive cycling tour through the Netherlands, they unexpectedly attended the dinner party after my thesis defence ceremony. Being a fresh PhD, I felt very honoured with his presence and his personal speech.

**Fig. 1:**
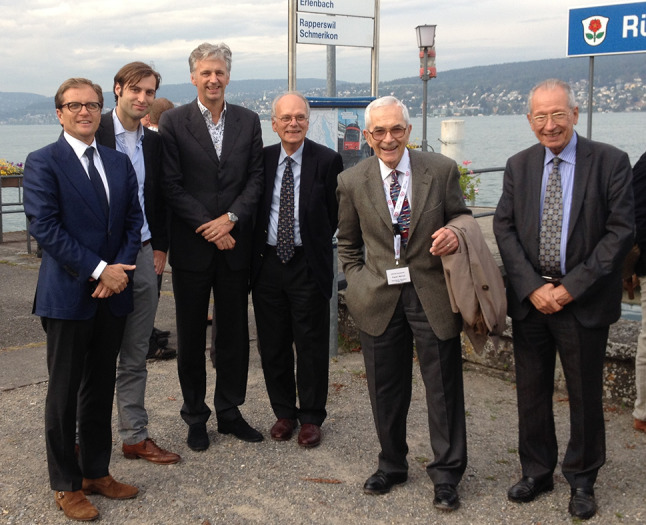
At the Arrhythmogenic Cardiomyopathy meeting in Zürich in 2016. *From right to left*: Guy Fontaine (France), Frank Marcus (USA), Richard Hauer, Peter van Tintelen, Thomas Mast and Maarten-Jan Cramer (all from Utrecht, the Netherlands)

While being a great scientist and physician, Marcus always remained a modest person. His sabbaticals in Paris and later in Bordeaux to learn from European research are examples: never ‘America first’, but with a strong belief in collaboration [3, 4]. ARVC studies in Utrecht since the late 90s promoted friendship with young Dutch PhD students. Marcus’ work facilitated our nationwide genotype-phenotype correlation studies and later the international collaboration-based risk stratification analysis. Collaboration of all Dutch university hospitals in ARVC resulted in a national registry, now the largest Arrhythmogenic Cardiomyopathy (ACM) registry in Europe (Fig. 1). With his discoveries and continuous support, Marcus became highly instrumental to our professional and personal careers. ARVC/ACM was the major subject in six PhD graduations in the Netherlands and more are expected! We will never forget our beloved friend.
